# Flux balance analysis with or without molecular crowding fails to predict two thirds of experimentally observed epistasis in yeast

**DOI:** 10.1038/s41598-019-47935-6

**Published:** 2019-08-14

**Authors:** Deya Alzoubi, Abdelmoneim Amer Desouki, Martin J. Lercher

**Affiliations:** Institute for Computer Science and Department of Biology, Heinrich Heine University, Universitätsstraße 1, Düsseldorf, D-40221 Germany

**Keywords:** Computer modelling, Epistasis

## Abstract

Computational predictions of double gene knockout effects by flux balance analysis (FBA) have been used to characterized genome-wide patterns of epistasis in microorganisms. However, it is unclear how *in silico* predictions are related to *in vivo* epistasis, as FBA predicted only a minority of experimentally observed genetic interactions between non-essential metabolic genes in yeast. Here, we perform a detailed comparison of yeast experimental epistasis data to predictions generated with different constraint-based metabolic modeling algorithms. The tested methods comprise standard FBA; a variant of MOMA, which was specifically designed to predict fitness effects of non-essential gene knockouts; and two alternative implementations of FBA with macro-molecular crowding, which account approximately for enzyme kinetics. The number of interactions uniquely predicted by one method is typically larger than its overlap with any alternative method. Only 20% of negative and 10% of positive interactions jointly predicted by all methods are confirmed by the experimental data; almost all unique predictions appear to be false. More than two thirds of epistatic interactions are undetectable by any of the tested methods. The low prediction accuracies indicate that the physiology of yeast double metabolic gene knockouts is dominated by processes not captured by current constraint-based analysis methods.

## Introduction

Epistasis measures the extent to which the consequences of a mutation in one gene depend on mutations in another gene^1^. Epistasis is said to be negative (aggravating) if the double mutant has lower fitness than expected, i.e., if its fitness is lower than the product of the single-mutant fitnesses; epistasis is called positive (alleviating) if the double mutant has higher fitness. Understanding the distribution of epistasis is fundamental to our understanding of gene function and interaction^2–4^. Epistasis is important for a wide range of theoretical issues in biology, including the evolution of sex^5,6^, speciation^7^, ploidy^8^, mutation load^9^, and genetic buffering^10^; epistasis is also fundamental to our understanding ofhuman disease^11,12^ and drug resistance^13^.

Epistasis can be assayed experimentally through the analyis of double gene knockouts^14–23^. However, such experiments are technically demanding, and the number of possible interactions grows quadratically with genome size. An attractive alternative to the generation of experimental knockouts for all possible gene combinations is the *in silico* prediction of double gene knockout effects. One approach towards the computational prediction of epistasis uses machine learning based on various experimentally observed gene and gene pair properties; Table 1 of ref.^24^ provides on overview over such predictions.

Here, we will focus instead on prediction methods based on *in silico* models of gene function, which are inherently more suited to generate increased biological understanding. Epistasis is a property of functional links between genes, not of individual genes. Thus, large-scale predictions of epistasis from first principles are only possible with computational models that account for functional connections between gene products. The best-studied complex biological system is metabolism. Excellent representations of metabolic networks have been compiled for several unicellular organisms such as *E*. *coli*^25^ and the baker’s yeast *Saccharomyces cerevisiae*^26^. So far, all attempts at genome-scale *in silico* epistasis prediction^27–34^ have used flux balance analysis (FBA), which maximizes the yield of biomass production in the wild-type and in the mutants^35,36^, or a variant of FBA that attempts to minimize the difference between wild-type and knockout distributions of metabolic reaction rates (minimization of metabolic adjustment, MOMA^37^).

## Previous in Silico Analyses of Epistasis

Several studies used these simulation methods to perform large-scale characterizations of epistasis *in silico*. Segrè *et al*. first used FBA to study the spectrum of epistatic interactions between metabolic genes in *S*. *cerevisiae*^27^. These authors introduced a new concept of epistasis between functional modules rather than between individual genes, intended to describe functional relationships among metabolic pathways. They found that modules interact with each other ‘monochromatically’, *i*.*e*., epistatic interactions between two specific modules are either largely positive or largely negative^27^. Examining the metabolic networks of *E*. *coli* and *S*. *cerevisiae*, He *et al*.^28^ found negative epistatic interactions largely among nonessential reactions with overlapping functions; in contrast, positive interactions were found predominantly between reactions without overlapping functions, and these were frequently essential^28^.

Snitkin *et al*.^29^ studied epistatic interactions between yeast gene deletions based on their influence on the reaction rates of individual enzymatic reactions. They found that gene pairs interact incoherently relative to different phenotypes, and that genes involved in many genetic interactions across multiple phenotypes tend to be highly expressed, to evolve slowly, and to be associated with human diseases^29^. Xu *et al*.^30^ compared epistatic interactions for different alleles of the same gene; alleles of different enzymatic activities were simulated by reducing the admissible flux (reaction rate) relative to the wild-type by a given percentage. They found that different alleles of the same gene typically interact with very different gene sets *in silico*; they argued that the distribution of the sign of epistasis in their simulations can speed up the purging of deleterious mutations in eukaryotes^30^. Finally, Barker *et al*.^31^ studied epistatic relationships between genes in various environments, finding that epistatic interactions can differ substantially between growth conditions and that the epistasis network structure differs fundamentally between condition-independent (stable) and condition-dependent interactions^31^.

## Relationship Between Predicted and Observed Epistasis

While the *in silico* analyses of epistatic landscapes summarized above purport to fundamentally advance our understanding of epistasis in nature, it is not clear that *in silico* and *in vivo* epistasis are correlated sufficiently on the genome-scale to allow such conclusions. Synthetic lethality – an extreme case of epistasis – was successfully predicted for some genes using FBA already in 2007; however, these authors could correctly predict only 7 out of 29 previously described synthetic lethals, corresponding to a recall of only 24%^32^. Two further studies in 2015 compared FBA predictions of synthetic lethality to experimental observations in yeast^38^ and *E*. *coli*^39^, confirming that only a minority of observed synthetic lethal interactions can be predicted successfully.

Several experimental platforms for the high-throughput detection of epistasis have been developed, among them synthetic genetic arrays (SGA)^15,23^, diploid-based synthetic lethality analyses with microarrays^16,19^, synthetic dosage-suppression and lethality screens^14,17,18^, and epistatic miniarray profiles^20–22^. The most comprehensive estimates of epistasis are available for the baker’s yeast *Saccharomyces cerevisiae*^23,33^, obtained through SGA. Szappanos *et al*.^33^ were the first to compare quantitative epistasis predictions from FBA and MOMA with high-throughput experimental data, examining 67,517 pairs of non-essential yeast genes (high-confidence empirical interactions from SGA). They also found that only a minority of empirically observed interactions can be successfully predicted. For negative epistatic interactions, at 45% precision (percentage of predicted interactions that are indeed experimentally observed), they obtained a recall (percentage of observed interactions that are correctly predicted) of 2.8%. While the recall can be increased to slightly above 4% by lowering the prediction threshold, this comes at the cost of many false positive predictions, associated with a drastic reduction of precision to below 6%. For positive interactions, Szappanos *et al*. obtained a recall of 12.9% at a precision of around 10%, which could not be improved much further by lowering the prediction threshold. Furthermore, the prediction quality could only be improved marginally by an automated model refinement procedure^33^. These results suggest that the physiological responses of yeast to double gene knockouts are not sufficiently captured by computational methods based on yield maximization such as FBA and MOMA. A later study that calculated epistasis from a new “function-loss cost” metric did not result in significantly improved predictions of the same data^34^.

## Constraint-based Modelling Strategies that Might Improve Prediction Accuracy

Why did the methods tested – FBA and MOMA – perform so poorly when predicting epistatic interactions? FBA captures epistasis based on the maximal biomass yield of the single and double mutants. MOMA assumes that the redistribution of reaction fluxes relative to the FBA wild-type solution is minimized upon the genetic perturbation^37^. Both FBA and MOMA predictions ignore the protein cost of enzymatic reactions, which arises from the necessary investment of cellular currencies, such as ATP and carbon, into enzyme production. Furthermore, it has been suggested that enzymes and the protein translation apparatus compete for the limited intracellular concentration space, a suggestion consistent with the observation that total cellular protein concentrations appear to be approximately constant across conditions^40^. In particular the latter constraint, summarized under the term (macro-) molecular crowding, has been explored in detail in the literature^41–43^. Instead of a largely arbitrary constraint on the uptake of a limiting nutrient, FBA models with molecular crowding limit cell growth by imposing a maximal mass concentration of enzymes, which in turn limits the total flux through the reactions the enzymes catalyze. Note that FBA and related constraint-based models do not consider internal metabolite concentrations explicitly, and thus FBA with molecular crowding methods calculate the enzyme concentration necessary for a given reaction flux *v* as [*E*] = *v*/*k*_*eff*_, with a constant effective rate constant that is often approximated through the enzyme turnover number *k*_*cat*_^41,43^.

Could molecular crowding be responsible for epistatic interactions? FBA considers different yields of pathways, but pathways also differ in their kinetics, such that the same overall flux may require much more protein investment in one pathway compared to an alternative pathway; such differences in pathway costs of fluxes are believed to be the origin of overflow metabolism^44,45^. Accordingly, the fitness effect of a non-essential enzyme knockout will depend not only on the stoichiometry of the catalyzed reaction (which is what FBA considers), but also on the enzyme’s kinetics (additionally considered by FBA with molecular crowding).

**Figure 1:**
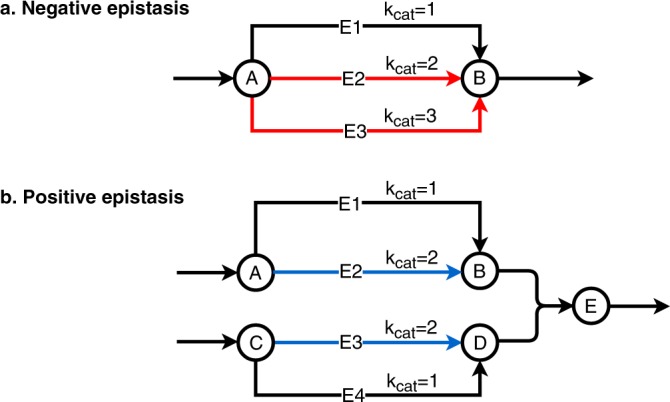
Illustrative examples of epistatic interactions that arise because of different enzymatic costs of pathways. (**a**) Negative epistasis between E2 and E3. (**b**) Positive epistasis between E2 and E3. The example assumes equal protein costs for all enzymes.

Two toy examples for positive and negative epistasis are given in Fig. 1. If multiple isoenzymes or pathways can convert metabolite A into B (Fig. 1a), then FBA will predict that the corresponding single and double knockouts are all without fitness effect. However, if the isoenzymes and pathways differ in the protein cost per catalyzed flux, then a double knockout involving the most efficient enzymes will result in a reduced total flux, unless protein investment into the remaining pathway is increased at the cost of reduced investment into other pathways that contribute to biomass. The least effective pathway is utilized only in the double knockout, and this will result in negative epistasis. Positive epistasis may arise, e.g., if two pathways are coupled by a downstream enzyme that jointly uses the products of both pathways as substrates (Fig. 1b). If there exists a catalytically less efficient alternative pathway for each of the two inputs, then the double knockout of the two efficient pathways will result – at identical protein investment – in a flux that is identical to the lower flux of the two single knockouts.

Previous applications of MOMA^37^ suffer from a second problem. FBA solutions are generally redundant, *i*.*e*., multiple flux distributions lead to the same biomass yield. Thus, the distance of the MOMA to the FBA flux distribution may depend strongly on the particular FBA solution returned by the numerical solver of the wild-type optimization problem. A straightforward possibility to rectify this problem is to use the wild-type flux distribution returned by parsimonious FBA (pFBA), which attempts to minimize protein investment at a given biomass yield^46^ and has been shown to perform well in predicting the effects of single gene knockouts^47^.

Here, to test if the poor performance of previous *in silico* predictions of epistasis^33,34^ can be improved by correcting the shortcomings discussed above, we compare the double gene knockout data for yeast in ref.^33^ to epistasis predictions from (i) FBA; (ii) FBA with molecular crowding, using two slightly different algorithms (MOMENT^43^ and ccFBA, see Materials and Methods); and (iii) MOMA starting from the pFBA solution for the wildtype flux distribution.

## Materials and Methods

### Experimental data

We used a high-confidence subset of *S*. *cerevisiae* epistasis data for metabolic genes identified in Szappanos *et al*.^33^. This data was generated using synthetic genetic array (SGA) screens. We excluded genes deemed to be essential by the metabolic model or blocked in the model. This resulted in 291 negative and 123 positive interactions among 71,994 non-essential gene pairs.

### Metabolic models and media

To model *S*. *cerevisiae* metabolism, we used the metabolic reconstruction yeast7.6 (https://sourceforge.net/projects/yeast)^48^. Following the authors of ref.^33^, we removed a set of genes from the metabolic model (CAN1, LYP1, URA3, LEU2, MET17) to mimic the strain background used in the experiments; we also used the same definition of the growth medium as in ref.^33^, which mimics the experimental conditions^33^. The resulting, strain-specific model encompasses 904 metabolic genes associated with 3,326 reactions.

We performed all simulations using *sybil*, a computer library for efficient modelling of metabolic networks^49^ in R^50^. Among other methods, *sybil* implements FBA, pFBA (minimization of total flux, MTF), MOMA, and diverse methods for genome-scale simulations of genetic perturbations.

### Flux balance analysis (FBA)

FBA identifies a flux distribution across the metabolic network that maximizes biomass yield under the constraints given by (i) the stoichiometry of enzymatic and transport reactions and (ii) lower and upper bounds on individual fluxes. The upper bounds on individual enzymatic fluxes are meant to reflect maximal enzyme capacity, and hence FBA could in principle also take enzyme kinetics into account; however, as enzyme capacities are generally unknown, the upper bounds are typically set to a value that is effectively infinite. Lower bounds on individual enzymatic reactions are set to zero for reactions deemed irreversible, and are (effectively) set to negative infinity for reversible reactions. Bounds on exchange reactions reflect maximal nutrient uptake or excretion rates. To estimate epistasis with FBA, we need to calculate the maximal biomass production yield of the double gene knockout, *v*_12_, and the two single gene knockouts, *v*_1_ and *v*_2_; in each case, all fluxes through reactions for which one of the knockouts is essential are forced to zero. We convert the biomass yield values to fitness estimates by dividing them by the wild-type biomass yield, *v*_*WT*_: *W*_*i*_ = *v*_*i*_/*v*_*WT*_. The fitness of the single and double mutants then allows the calculation of epistasis as^33^:

1 $$\varepsilon :={W}_{12}-{W}_{1}\times {W}_{2}$$

### Minimization of metabolic adjustment (lMOMA)

Minimization Of Metabolic Adjustment (MOMA) is an extension of FBA for the prediction of flux distributions in gene knockouts. MOMA employs quadratic programming to identify the closest point (in terms of its Euclidean distance) in the permissible flux space of the knockout to the wild-type flux vector^37^. Previous applications of MOMA to epistasis predictions minimized the distance to an arbitrary FBA solution returned by the linear solver of the FBA problem^33^. As FBA flux distributions are highly degenerate, we instead use the parsimonious FBA (pFBA or minimal total flux, MTF) solution to the wild-type problem^46^, which should lead to biologically more relevant results^47^. Following previous applications^33^, we minimize the Manhattan rather than Euclidean distance between wild-type and knockout flux distributions, which results in a linear optimization problem (lMOMA). As for FBA, epistasis was then estimated from the difference between the double knockout fitness and the product of the single knockout fitnesses (Eq. (1)).

### Metabolic modelling with enzyme kinetics (MOMENT)

MOMENT^43^ is an algorithm for performing FBA with molecular crowding^41,42^. MOMENT extends FBA by adding a global constraint on the total mass concentration (assumed to be proportional to volume concentration) of enzymes:

2 $${\sum }_{i}[{E}_{i}]{m}_{i}\le C,$$

where the sum runs over all enzymes (or enzyme complexes) *i*, [*E*_*i*_] is the molar concentration of enzyme *i* per gram dry weight,*m*_*i*_ is the molar mass of the enzyme, and *C* is an upper limit on the total enzyme mass per gram dry weight. The limit imposed by Eq. (2) replaces the constraint on nutrient uptake on the rate of biomass production. The authors of ref.^43^ set this limit to *C* = 0.27 (g enzymes/gDW) based on a fit between observed and predicted growth rates, suggesting that metabolic enzymes are responsible for about half of the total protein mass^43^. Note, however, that due to the linearity of the biomass production rate in *C*, *C* cancels in the ratio of knockout/wildtype biomass reaction fluxes, and hence its numerical value has no influence on the epistasis predictions according to Eq. (1). Note that in FBA with molecular crowding, the maximal biomass fluxes *v*_*WT*_, *v*_1_, *v*_2_, and *v*_12_ represent maximal growth rates rather than yields. As originally published, MOMENT is parameterized only for *E*. *coli*. Here, we use a re-implementation in the ccFBA package that includes a parameterization for *S*. *cerevisiae* (see next subsection).

### Cost-constrained FBA (ccFBA)

ccFBA^51^ is a general implementation of FBA with molecular crowding that largely implements the MOMENT algorithm^43^, but improves on MOMENT by explicitly considering multifunctional enzymes. ccFBA is implemented in R^50^ and builds on the *sybil* package^49^; it is distributed on CRAN (https://cran.r-project.org) and has briefly been described in ref.^52^. We replaced the iMM904 yeast model distributed with ccFBA with the yeast 7.6 model adapted to the experimental data (see above). The resulting ccFBA model contains experimental *k*_*cat*_ values for 535 enzymes; for the remaining enzymes, we use the median of the 535 known values^51^, *k*_*cat*,*med*_ = 11.5. The same model and parameters were used for the MOMENT (see previous subsection). The proportion of biomass devoted to metabolic enzymes was set to *C* = 0.27 as in MOMENT^43^, and epistasis was calculated accordingly.

## Results and Discussion

### Predicted interactions differ substantially between methods

For each pair of non-essential genes contained in the metabolic model, we calculated Epistasis (Eq. (1)) based on four methods: (i) standard flux balance analysis^35,36^ (FBA); (ii) a linear version of minimization of metabolic adjustment^37^ that finds the knockout flux distribution most similar to the pFBA prediction for the wildtype flux vector (lMOMA); (iii) metabolic modelling with enzyme kinetics^43^, an implementation of FBA with molecular crowding that approximately accounts for enzyme kinetics (MOMENT); and (iv) a modified implementation of MOMENT with a more realistic consideration of multifunctional enzymes^51,52^ (ccFBA). To obtain an overview over the differences between the tested methods, we first classified gene pairs into those showing negative epistasis (ε ≤ −0.0001), positive epistasis (ε ≥ +0.0001), or no epistasis (|ε| < 0.0001).

**Figure 2:**
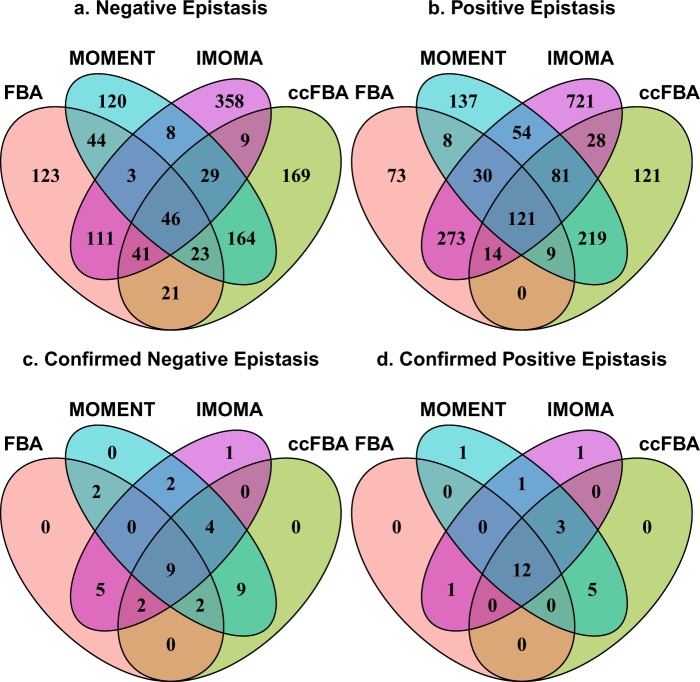
Venn diagrams showing the overlap of negative (**a**,**c**) and positive (**b**,**d**) epistasis predictions by the four methods. Panels (a) and (b) show total predictions. Panels (c) and (d) show only those predictions confirmed by the high-confidence set of experimental epistasis estimates.

The Venn diagrams in Fig. 2a,b summarize the sets of gene pairs that show negative and positive epistasis, respectively, according to the four methods. 46 negative and 121 positive interactions are predicted jointly by all four methods. The consideration of molecular crowding seems to have a strong effect on the epistasis predictions, as the results seem to fall into two clusters. 49% of negative and 83% of positive epistasis predictions by FBA are also predicted by lMOMA, while 60% of negative and 65% of positive interactions predicted by MOMENT are also predicted by ccFBA; the remaining pairs of methods show much smaller agreement (Fig. 2a,b). The numbers of interactions predicted uniquely by a single method differ substantially: While FBA predicts only 196 genetic interactions not predicted by any of the other methods, MOMENT makes 257 unique predictions, ccFBA makes 290 unique predictions, and lMOMA makes 1079 unique predictions.

### Epistasis predictions show little overlap with experimentally observed epistasis

The large numbers of unique predictions of individual methods could potentially indicate that each method captures distinct aspects of physiological responses to the knockouts. To test this possibility, we compared the epistasis predictions by the three methods to the high-confidence experimental epistasis data set provided by Szappanos *et al*.^33^. Figures 2c,d show Venn diagrams that compare the numbers of correctly predicted experimentally observed epistatic interactions between the four metabolic simulation methods. Only a small fraction of the predicted interactions are confirmed by the data in each case. Not surprisingly, the most reliable predictions are those that are jointly made by all four simulation methods (9 correct out of 46 joint predictions of negative epistasis, i.e., a precision of 9/46 = 19.6%; and 12 correct out of 121 joint predictions of positive epistasis, i.e., a precision of 12/121 = 9.91%). In contrast, only 3 out of 1822 genetic interactions uniquely predicted by one of the four methods (0.2%) are confirmed by the experiments.

The negative interactions jointly predicted by MOMENT and ccFBA are also confirmed in 24 out of 262 cases (9.2%), indicating that predictions based on the concept of molecular crowding that are robust to changes in method details tend to be more reliable than those that are not. These predictions include 21 confirmed cases not predicted by FBA (with a total of 33 confirmed predictions), indicating that combining standard FBA with MOMENT/ccFBA may improve the recall achievable in FBA predictions compared to FBA alone.

**Figure 3:**
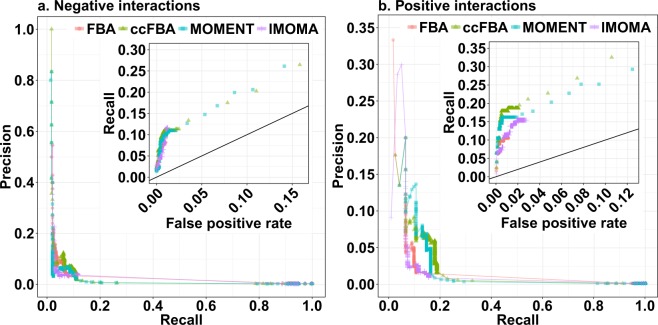
The accuracy of the four prediction methods for (**a**) negative and (**b**) positive epistatic interactions. The outer panels show precision (fraction of predictions that are confirmed by the experimental data) *vs*. recall (fraction of experimentally observed interactions that are predicted correctly), while the insets show a detail of the receiver operator characteristic (ROC) curve, tracing the dependence of recall on the false positive prediction rate (FPR = 1 – specificity, the fraction of predicted epistasis cases that are not confirmed by the experimental data).

The cutoff of |ε| = 0.0001 for epistasis used to select the predicted interacting gene pair sets in Fig. 2 was chosen largely arbitrarily. Figure 3 shows the influence of other cutoffs for the simulated epistasis scores ε on prediction accuracy. Each data point in the main figures shows a combination of recall (the fraction of experimentally observed high-confidence interactions that is predicted *in silico*) and precision (the fraction of predicted interactions that is confirmed by experimental data) at a given cutoff |ε| by the method indicated by the colour. Higher cutoffs mean that only predictions of strong epistasis are deemed reliable, and lead to lower recall and generally to higher precision; lower cutoffs deem more epistasis predictions reliable and consequently lead to higher recall but generally lower precision. The data points in the insets (a detail of the receiver operator characteristic, ROC) represent the same predictions, but report the false positive rate (the fraction of *in silico* predictions that are not confirmed by the data) instead of the precision. Higher cutoffs |ε| lead not only to lower recall, but also to lower false positive rates; lower cutoffs increase both recall and false positive rate. The distance of data points to the diagonal (which represents random “predictions”) indicate the accuracy of predictions at this cutoff, where prefect predictions would lie in the top left corner.

For negative interactions, all four methods show a similar relationship between recall and precision (Fig. 3a) and between recall and false positive rate (Fig. 3a, inset). For positive interactions, these relationships are similar between FBA and lMOMA on one hand and between the methods accounting for molecular crowding on the other. If accepting precision values below 10%, MOMENT and ccFBA achieve higher recall values than the two alternative methods (Fig. 3b).

Importantly, even with the most generous cutoffs, the highest recall reachable by any of the methods is at or below 33%. Thus, two thirds of experimentally observed epistatic interactions are not detectable by any of the constraint-based methods tested, regardless of how many false positives we are willing to accept. To achieve recall values above 20%, we have to accept false positive rates of more than 8% for negative and 2% for positive interactions; given the high number of comparisons made (71,994 in the dataset used here), this means that true predictions of epistasis are drowned in a sea of false predictions. At a more reasonable false positive rate of 1%, the highest achievable recall values are around 12% for negative interactions and 19% for positive interactions.

### Prediction accuracy is equally low for synthetic lethals

To predict epistasis scores for viable double mutants, we need to calculate fitness values quantitatively for the single and double knockouts. It is conceivable that the underwhelming performance of constraint-based methods to predict genetic interactions (Figs 2, 3) is due to this requirement of quantitative predictions; indeed, a previous study showed that quantitative predictions of non-lethal gene knockout fitness values correlate only weakly with experimental observations^53^. In contrast, the strength of constraint-based methods may lie more in qualitative predictions: FBA has been demonstrated to accurately predict gene essentiality, i.e., genes whose knockout is lethal^54,55^. The likely reason is that knockout lethality often arises from the inability to produce a biomass component without the knocked out reaction, i.e., from an effect of the knockout on metabolic network topology rather than on kinetics, regulation, or biomass yield. Thus, it might be reasonable to expect that constraint-based methods also perform well when predicting synthetic lethals, i.e., gene pairs where the single mutants are viable but the double mutant is not. In disagreement with this expectation, previous studies showed recall values below 25% for the FBA prediction of synthetic lethals^32,38^. However, these observations were based on the analysis of small numbers of experimentally confirmed synthetic lethals drawn from diverse studies, and thus it seems advisable to compare model predictions of synthetic lethality to a systematic, genome-wide screen of metabolic genes.

To identify pairs of synthetic lethal genes in the raw data from ref.^33^, we selected non-essential gene pairs with experimentally confirmed negative epistasis (ε < −0.08, see ref.^23^) and with very low double mutant fitness (*f* < 0.2). Only 146 out of a total of 207,060 non-essential gene pairs represented in the model and assayed by Szappanos *et al*.^33^ were labeled as synthetic lethal according to this definition.

When using the same cutoffs (ε < −0.08 and *f* < 0.2) for the computational epistasis predictions, we recover only 4 (FBA), 4 (lMOMA), 0 (ccFBA), and 0 (MOMENT), respectively, of the experimentally confirmed synthetic lethal pairs. This corresponds to recall values below 3%. For FBA, lMOMA, and MOMENT, recall cannot be improved by choosing less stringent cutoffs, as long as we require negative epistasis. For ccFBA, we can obtain 6 true positive predictions if we relax the double mutant fitness cutoff to *f* < 0.55 while requiring negative epistasis (ε < −0.0001). These findings confirm the earlier results on smaller datasets of synthetic lethals^32,38^: constrained based methods appear no better at predicting synthetic lethality than at predicting epistasis in general.

## Conclusions

While the incorporation of a constraint for molecular crowding in MOMENT and ccFBA added a small number of correct epistasis predictions to those obtained using standard FBA and lMOMA, the most important conclusion that can be drawn from the above analyses is a sobering one: We still fail to predict two thirds of experimentally observed epistatic interactions, regardless of the constraint-based method and the cutoffs used. Thus, neither the inclusion of molecular crowding in MOMENT and ccFBA nor the use of a more realistic wild-type flux distribution in lMOMA led to a substantial improvement over the previously reported failure of FBA to predict a majority of experimentally observed interactions^33^.

The essence of FBA with molecular crowding, as implemented in MOMENT and ccFBA, is the incorporation of a tradeoff: the expression of one pathway reduces the cellular resources available for other pathways. This interdependence between pathways in terms of available resources may underlie at least some epistatic interactions, and may hence contribute to explaining why these methods were able to expand the set of correctly predicted interactions (Fig. 2). Both methods, which assume enzyme turnover rates that are independent of metabolite concentrations, provide only rough approximations of the cellular constraints related to enzyme kinetics. Their approximate nature may be reflected in the higher reliability of epistasis predictions made jointly by both methods. It is conceivable that a substantial fraction of observed epistatic interactions can only be understood through a more detailed consideration of reaction kinetics and the associated cellular investment into enzymes. In this context, we need to emphasize that the yeast model employed here contains known enzyme turnover numbers (*k*_*cat*_) for only 535 out of 4,594 protein-associated reactions, and it is conceivable that an improved parameterization may lead to improved prediction accuracy. However, none of the tested methods could correctly predict synthetic lethal interactions, which in most cases probably arise from changes in network topology rather than from enzyme kinetics; this failure suggests that the problem is more fundamental.

A second potential explanation for the observed underperformance of constraint-based methods is the influence of regulatory feedbacks. Regulatory interactions evolved in the ancestors of the wild-type strain as responses to environmental conditions. Changes in metabolite concentrations resulting from the knockouts may be mis-interpreted by the cell’s regulatory system as environmental cues, and may thus lead to regulatory responses that cause suboptimal metabolic network usage. Such “inappropriate” regulatory responses might lead to large discrepancies between mutant physiology and predictions by optimization-based methods ignorant of regulatory circuits.

If true, the hypothesis advanced in the last paragraph has important implications not only for the utilization of constraint-based methods to predict experimentally observed epistasis, but also for the biological interpretation of double knockout mutant physiology. If the observed effects of double knockouts are in large part due to regulatory responses, they may provide little information on the interaction of the gene products in wildtype physiology.

## Supplementary Information

Supplementary Dataset 1

Supplementary Dataset 2

## Data Availability

The data summarized in Figs 2 and 3 (Dataset 1) and the synthetic lethal data (Dataset 2) are provided as Supplementary Datasets. The empirical data, the modified yeast7.6 metabolic model, and the turnover numbers (*k*_*cat*_) and molecular weights used as input to MOMENT and ccFBA can be found on github at https://github.com/deyazoubi/Epistasis-; an overview over the individual files is given in the Readme file.
